# Abnormal T2 mapping cardiovascular magnetic resonance correlates with adverse clinical outcome in patients with suspected acute myocarditis

**DOI:** 10.1186/s12968-017-0350-x

**Published:** 2017-03-29

**Authors:** Maximilian Spieker, Sebastian Haberkorn, Mareike Gastl, Patrick Behm, Stratis Katsianos, Patrick Horn, Christoph Jacoby, Bernhard Schnackenburg, Petra Reinecke, Malte Kelm, Ralf Westenfeld, Florian Bönner

**Affiliations:** 1 Department of Cardiology, Pulmonology and Vascular Medicine, Heinrich Heine University Düsseldorf, Moorenstraße 5, 40225 Düsseldorf, Germany; 2 Philips Healthcare, Hamburg, Germany; 3 Institute of Pathology, Heinrich Heine University, Duesseldorf, Germany; 4 CARID, Cardiovascular Research Institute Düsseldorf, Duesseldorf, Germany

**Keywords:** Myocarditis, T2 Mapping, Prognostic implication

## Abstract

**Background:**

While most patients recover from suspected acute myocarditis (sAMC) some develop progressive disease with 5-year mortality up to 20%. Recently, parametric Cardiovascular Magnetic Resonance (CMR) approaches, quantifying native T1 and T2 relaxation time, have demonstrated the ability to increase diagnostic accuracy. However, prognostic implications of T2 values in this cohort are unknown. The purpose of the study was to investigate the prognostic relevance of elevated CMR T2 values in patients with sAMC.

**Methods and Results:**

We carried out a prospective study in 46 patients with sAMC defined by current ESC recommendations. A combined endpoint was defined by the occurrence of at least one major adverse cardiac event (MACE) and hospitalisation for heart failure. Event rate was 24% (*n* = 11) for 1-year-MACE and hospitalisation. A follow-up after 11 ± 7 months was performed in 98% of the patients. Global T2 values were significantly increased at acute stage of disease compared to controls and decreased over time. During acute disease, elevated global T2 time (odds ratio 6.3, *p* < 0.02) as well as myocardial fraction with T2 time >80 ms (odds ratio 4.9, *p* < 0.04) predicted occurrence of the combined endpoint. Patients with clinical recovery revealed significantly decreased T2 relaxation times at follow-up examinations; however, T2 values were still elevated compared to healthy controls.

**Conclusion:**

Assessment of myocardial T2 relaxation times at initial presentation facilitates CMR-based risk stratification in patients with acute myocarditis. T2 Mapping may emerge as a new tool to monitor inflammatory myocardial injuries during the course of disease.

## Background

Myocarditis is a common cardiac disease with a prevalence of 22 per 100.000 people worldwide [1]. However, the diagnosis remains challenging due to its variable clinical presentation and individual shortcomings of diagnostic tools. Myocarditis has been reported in up to 20% of sudden cardiac deaths among young adults and is regarded as precursor of dilated cardiomyopathy [2, 3]. In the light of a highly variable clinical presentation ranging from full recovery in the majority of patients to progressive heart failure in some patients, diagnostic accuracy and individual risk stratification is of prime importance. Patients with severe forms of myocarditis have a 5-year mortality of almost 20% [4].

Recent recommendations highlight the need for new diagnostic criteria in patients with suspected acute myocarditis (sAMC) and endorse coronary angiography as well as endomyocardial biopsy (EMB) during routine diagnostic workup [5, 6]. Besides EMB, Cardiovascular Magnetic Resonance (CMR) emerged as a key non-invasive tool for diagnosis of myocarditis [7]. Recently, parametric CMR approaches quantifying native T1 and T2 relaxation time, have increased CMR-based diagnostic accuracy for sAMC [8–13]. Additionally, increased myocardial T2 values were shown to correlate with edema [14] and myocardial inflammation in EMB [8, 10].

Recently, we demonstrated increased T2 values in patients with biopsy-proven acute myocarditis (bpAMC) compared to age and gender matched controls [10, 15]. Furthermore, we proposed a diagnostic approach quantifying the extent of myocardium with pathologically elevated T2 time expressed as percentage of whole myocardium, thus taking accumulating regional inflammation into account. This strategy improved CMR-based diagnosis of bpAMC. So far, prognostic implications of abnormal elevated T2 values with respect to clinical and functional recovery are unknown.

Until now, several predictors of poor outcome have been identified, including reduced left ventricular ejection fraction (LVEF) [16], advanced New York Heart Association (NYHA) class, grade of histopathological forms of tissue damage [17] and presence of Late Gadolinium Enhancement (LGE) [4].

The aim of the presented study was to prospectively recruit patients with sAMC and conduct CMR and EMB as systematic as feasible in order to assess the prognostic value of T2 Mapping in this patient cohort. We hypothesized that T2 Mapping facilitates risk stratification in patients referred for CMR workup of sAMC. In particular, we sought to evaluate whether T2 Mapping provides additional prognostic information over conventional CMR parameters and identifies patients at high-risk for following adverse cardiac events.

## Methods

### Study population

The ethical board of Heinrich-Heine University Düsseldorf approved the present study (application number 4307). All participants signed informed consent. The study complies with the declaration of Helsinki.

Forty-six patients meeting inclusion criteria of sAMC according to ESC guidelines were prospectively enrolled between 2013 and 2016: clinical symptoms <14 days (dyspnoea, chest pain, fatigue) and one additional diagnostic criterion in terms of ECG abnormalities (ST-changes, conduction defects), hsTNT-elevation or new global/regional wall motion abnormalities and exclusion of coronary artery disease.

Exclusion criteria of the study were 1) coronary artery disease (coronary stenosis >50% proven by angiography 2) pre-existing other cardiac disease that could explain symptoms and 3) contraindications against CMR.

All patients underwent CMR between day 0 and day 5 after initial presentation at hospital. Right ventricular EMB was conducted whenever feasible (*n* = 40, 72 ± 12 h after presentation).

Patients were scheduled after informed consent for a follow-up assessment 6 to 18 months after the initial hospitalisation. Left ventricular systolic function at follow-up was assessed by CMR or 3D-echocardiography. Sixty age, sex and cardiovascular risk factor -matched volunteers served as controls.

### CMR imaging and analysis

CMR was performed using a 1.5 tesla scanner (Achieva, Philips, Best Netherlands) with a 32-channel phased array coil. After scout and reference scans, CINE-loops in continuous short axis slices covering the whole ventricle were analyzed to calculate left ventricular function and volumes.

*For edema imaging*, a T2-weighted TSE-STIR sequence was used and T2 Mapping was conducted with a GRASE sequence in three short axis slices (basal, midventricular and apical). This sequence combines the TSE and echo-planar imaging methods by using a train of refocusing 180° pulses and an odd number of additional gradient echoes for each spin echo. This sequence was used with cardiac triggering and respiration navigator gating with the following parameters: TR = 1 RR interval, number of echo images = 15, echo spacing 10 ms, leading to an echo train of 150 ms, number of gradient echoes for segmented acquisition = 3 (EPI factor), FA = 90°, spatial resolution: 2 × 2 × 10 mm^3^, parallel imaging (SENSE) with an accelerating factor of 2, k-space data acquired with Cartesian encoding scheme. For blood saturation a double inversion (black-blood) pulse was used [14].

*Late Gadolinium Enhancement (LGE) imaging* was performed eight to ten minutes after gadolinium contrast injection (ProHance®, Bracco Imaging, 0.2 mmol/kg) using a 3-dimensional–gradient spoiled turbo fast-field-echo sequence with a non-selective 180° inversion-recovery pre-pulse triggered to end-diastole acquired in the short axis, 4–3- and 2-chamber view to cover the whole ventricle.

*For post-processing*, left ventricular function and LGE were assessed off-line using a commercial software (Extended Workspace, Philips Healthcare). LGE was evaluated by visual assessment and assignment of certain myocardial parts according to the 17-segment model of the AHA [18].

T2 maps were generated according to Bönner et al. [15]. T2 maps were generated off-line using software based on the graphical programming language LabVIEW (National Instruments, Austin, TX). An exponential decay curve was fitted to the intensity decline of each pixel within the images obtained from the multi echo sequence. We used a 2-parameter (amplitude and damping) fit model with a constant off-set. The bias was calculated from the noise of all echo images and assumed to be constant, so that the problem could be linearized and the regression coefficient (R^2^) could be used as a goodness-of-fit parameter in order to exclude accidental values. If R^2^ was not within a tolerance interval chosen to be 0.7-1, the corresponding T2 value was not considered for further calculations. The resulting T2 constants were colour-coded using the spectral look-up table. Endo- and epimyocardial region of interests (ROI) were drawn manually in the first echo image of the echo-train.

The myocardial ROI was segmented according to the AHA 17-segment model [18] and T2 values were calculated for 16 segments (segment 17 was not considered). In order to detect regional edema more reliably we quantified the myocardial fraction with T2 time over several cut-offs. ROC analysis suggested that a selected cut-off over 80 ms was the best parameter to distinguish between biopsy-proven inflamed myocardium and healthy myocardium. This cut-off was determined in a previous study including 26 patients with bpAMC and 60 healthy controls [10]. The myocardial fraction exceeding 80 ms was quantified and expressed as percentage of the whole myocardium and graphically highlighted in white. To avoid spill over of high T2 values due to epicardial fat and endocardial slow flow artefacts a T2 time limit of 110 ms was chosen in the final absolute T2 time and fractional quantification.

### Endpoints and clinical follow-up

Patients were followed over time and a combined clinical endpoint was defined as a composite of major adverse cardiac events (MACE) and hospitalisation due to heart failure. MACE was defined as a composite of all-cause death, cardiac death, cardiac transplantation and ventricular assist device implantation. The length of follow-up was determined by occurrence of an endpoint or the last clinical follow-up examination.

### Statistical analysis

Statistical analysis was performed with Sigma Stat2010 (Systat GmbH) and GraphPad Prism 7. Unless otherwise stated, data are presented as mean value ± standard deviation. Data were statistically analysed by the paired or unpaired Student’s *t*-test. Welch’s correction was used when unequal SD was assumed. Bonferroni correction was applied for multiple comparisons. Fisher’s exact test was used to examine the significance of the association between two kinds of classification. Receiver operator characteristic (ROC) analysis was performed to generate threshold values with respect to optimal sensitivities, specificities and areas under the curve (AUC). Kaplan-Meier curves were calculated for visualizing cumulative event free survival of patients with global and regional T2 time exceeding several cut-offs. P-values below 0.05 were assumed to be significant.

## Results

### Baseline patient characteristics

A total of 46 patients matched inclusion criteria. Table 1 shows baseline patient characteristics. Positive troponin (troponin >14.0 ng/ml) was detected in 83% with a mean value of 375 ± 512 ng/ml. The majority (57%) suffered from chest pain on admission and 43% of patients complained about severe dyspnoea (NYHA class III to IV).

**Table 1:** Classification of sAMC patients according to current recommendations

	sAMC
	*n* = 46
Clinical Presentation	
Acute Chest Pain	26 (57%)
New-onset or worsening of dyspnoea at rest or exercise:	34 (74%)
NYHA I	12 (26%)
NYHA II	14 (30%)
NYHA III	14 (30%)
NYHA IV	6 (13%)
Arrhythmia symptoms; Palpitations; Syncope	9 (20%)
Fatigue	33 (72%)
Diagnostic Criteria	
Current Infection	36 (78%)
Hs-Troponin (ng/ml)	375 ± 512
Hs-Troponin >14 ng/ml	38 (83%)
Suspicious ECG (%)	32 (70%)
Functional and structural abnormalities on cardiac imaging (CMR/Echo)	46 (100%)

*Abbreviations*: *sAMC* suspected acute myocarditis, *ECG* Electrocardiogram, *Echo* Echocardiography, *NYHA* New York Heart Association, *CMR* Cardiovascular Magnetic Resonance

Values represent mean ± standard deviation or percentage

Forty-three patients underwent coronary angiography to exclude coronary artery disease (CAD). Another three patients (age < 35 years) refused invasive coronary assessment. Forty patients underwent EMB during initial workup. Within those 40 patients, 22 were characterized as having bpAMC.

Initial CMR assessment demonstrated significantly reduced LVEF in patients with sAMC compared to controls (sAMC (*n* = 46) vs. controls (*n* = 60): 42 ± 15% vs. 62 ± 8%; *p* < 0.001). LGE was present in 80% of all sAMC patients. T2 time was significantly elevated in patients with sAMC (sAMC (*n* = 46) vs. controls (*n* = 60): 68.1 ± 5.8 ms vs. 60.0 ± 4.2 ms; *p* < 0.001). (Table 2)

**Table 2:** Characteristics of sAMC patients and healthy controls

	controls	sAMC	*p*-value
	*n* = 60	*n* = 46	
Female (*n*)	17 (28%)	13 (28%)	0.993
Age (years)	43 ± 12	41 ± 16	0.783
20–34 (*n*)	23 (38%)	17 (37%)	0.886
35–49 (*n*)	23 (38%)	16 (35%)	0.710
> 50 (*n*)	14 (23%)	13 (28%)	0.568
BMI (kg/m^2^)	24 ± 3	25 ± 4	0.819
Creatinine (mg/dl)	n.a.	1.2 ± 1.9	n.a.
CMR Parameters			
LVEF %	62 ± 8	42 ± 15	<0.001
LVEDVi (ml/m^2^)	75 ± 18	80 ± 19	0.084
LVEDDi (ml/m^2^)	28±5	32±5	0.046
Presence of LGE (n)	0	37 (80%)	<0.001
T2 Ratio > 1.9 (n)	7 (12%)	20 (43%)	<0.001
Global T2 Time (ms)	60.0 ± 4.2	68.1 ± 5.8	<0.001
Fraction with T2 Time >80 ms (%)	4.1 ± 3.0	17.7 ± 11.5	<0.001

*Abbreviations*: *sAMC* suspected acute myocarditis, *BMI* Body Mass Index, *CMR* Cardiovascular Magnetic Resonance, *LVEF* Left ventricular ejection fraction, *LVEDVi* Left Ventricular End Diastolic Volume index, *LVEDDi* Left Ventricular End Systolic Diameter index

Values are mean ± standard deviation or percentage

### Clinical follow-up

Forty-five patients (98%) underwent follow-up examination after a mean interval of 11 ± 7 months. Eleven patients (24%) reached the combined endpoint: One patient died, one patient underwent heart transplantation and three patients received ventricular assist device implantation due to severe, therapy-refractory heart failure. Six patients were re-admitted to hospital for decompensating heart failure. Thirteen patients (29%) showed persistent left ventricular dysfunction. Mean LVEF at follow-up was 51 ± 13% with a mean absolute increase in LVEF of 10 ± 13%.

### Predictors of clinical outcome

**Fig. 1:**
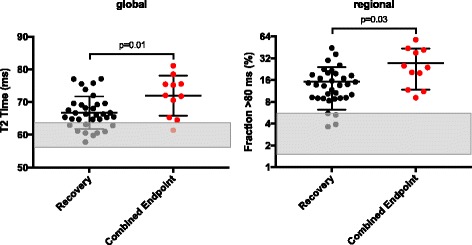
Predictive value of global and regional T2 time. Displayed are global T2 values on the left and regional T2 values with respect to area fraction exceeding 80 ms on the right. Patients having experienced MACE or were admitted to hospital due to heart failure are coloured in red. Reference range (mean ± SD) of global and regional T2 values for healthy controls are given as grey bars. Initial global T2 time of patients who experienced endpoint (*n* = 11) was 71.8 ± 5.7 ms while it was 66.8 ± 4.9 ms in those patients who did not (*n* = 45) (*p* = 0.01). In fact, the myocardial fraction with abnormal T2 time at first presentation was larger in patients who reached endpoint (combined endpoint: 27.5 ± 14.9% vs. no endpoint: 15.1 ± 8.7%. *p* = 0.03). Abbreviations: Fraction >80 ms = Percentage of myocardial fraction with T2 time >80 ms

**Fig. 2:**
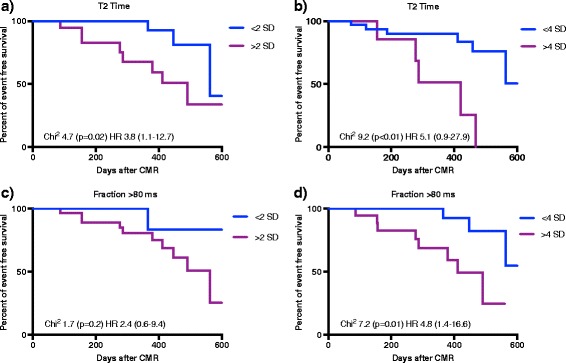
Kaplan-Meier Survival Curves for combined endpoint. Kaplan-Meier Survival Curves displaying event free survival according to **a** T2 Time <2 SD/>2 SD, **b** T2 Time <4 SD/>4 SD, **c** Fraction >80 ms <2 SD/>2 SD and **d** Fraction >80 ms <4 SD/>4 SD. Abbreviations: Fraction >80 ms = Percentage of myocardial fraction with T2 time >80 ms, SD = Standard deviation

Patients who experienced the combined endpoint displayed increased global and regional T2 values at first CMR compared to those who did not (combined endpoint (*n* = 11) vs. clinical recovery (*n* = 34), global T2 time: 71.8 ± 5.7 ms vs. 66.8 ± 4.9 ms, *p* = 0.01; fraction with T2 time >80 ms: 27.5 ± 14.9% vs. 15.1 ± 8.7, *p* = 0.03) (Fig. 1). At initial presentation, elevated global T2 time as well as extent of myocardium with T2 time above 80 ms predicted occurrence of the combined endpoint (Table 3). Kaplan-Meier survival curves generated for the combined endpoint are displayed in Fig. 2. Patients with global T2 time >4 SD represented a subgroup with elevated risk corresponding to a cumulative event rate of 55%. In contrast, patients with global T2 time <2 SD had a cumulative event rate of 14%.

**Table 3:** Predictors of adverse clinical outcome. Clinical presentation, EMB results and CMR parameters related to an adverse clinical outcome

	Recovery	Combined Endpoint	*p*-value	OR (CI 95%)
	(*n* = 34)	(*n* = 11)		
Age (years)	32.8 ± 14.7	48.4 ± 13.2	0.05	
male	24 (71)	5 (45)	0.16	2.9 (0.86–1.25)
Clinical Presentation				
Chest Pain	21 (62)	5 (45)	0.49	0.5 (0.13–1.83)
Palpitations	3 (9)	4 (36)	0.05	5.9 (1.27–26.33)
Fatigue	25 (74)	8 (73)	0.99	0.9(0.24–3.39)
Dyspnoea (NYHA Class)				
III	8 (24)	6 (55)	0.07	3.9 (1.04–16.96)
IV	4 (12)	2 (18)	0.62	1.7 (0.28–8.62)
Blood Testing				
Troponin (ng/ml)	363 ± 8135	103 ± 143	0.19	
BNP (pg/ml)	4046 ± 8135	3636 ± 2497	0.91	
EMB Results				
Imflammation on EMB	15 (54)	5 (50)	0.99	1.0 (0.29–4.21)
Presence of virus genome	13 (52)	8 (73)	0.29	2.5 (0.57–9.91)
CMR Parameters				
LVEF (%)	44 ± 13	33 ± 14	0.02	
LVEF <30%	7 (21)	7 (64)	0.02	6.8 (1.56–24.12)
Presence of LGE	26 (76)	10 (91)	0.25	4.2 (0.62–49.34)
T2 Ratio >1.9	14 (41)	5 (45)	0.99	1.2 (0.31–4.09)
Global T2 Time (ms)	66.8 ± 4.9	71.8 ± 5.7	0.01	
Global T2 Time, >2SD	12 (35)	8 (73)	0.04	4.9 (1.1–18.9)
Global T2 Time, >4SD	4 (12)	5 (45)	0.02	6.3 (1.2–24.9)
Fraction with T2 Time >80 ms (%)	15.1 ± 8.7	27.5 ± 14.9	0.03	
Fraction with T2 Time >80 ms, >2SD	23 (68)	10 (91)	0.24	4.7 (0.7–56.2)
Fraction with T2 Time >80 ms, >4SD	12 (35)	8 (73)	0.04	4.9 (1.1–18.9)

*Abbreviations*: *Fraction >80 ms* Percentage of myocardial fraction with T2 time >80 ms, *BNP* Brain Natriuretic Peptide, *EMB* Endomyocardial Biopsy, *LVEF* Left ventricular ejection fraction, *LGE* presence of Late Gadolinium Enhancement, *T2w Imaging* increased T2 ratio >1.9 (T2 weighted Imaging)

Likewise, reduced left ventricular function at initial presentation was associated with an unfavourable outcome: Seven patients (64%) who experienced the combined endpoint presented with severely reduced LVEF (LVEF <30%) (odds ratio 6.8, *p* < 0.02). In contrast 7 patients (21%) of those who recovered showed severely reduced LVEF at first presentation (Table 3). Moreover, initial impairment of LVEF was associated with an abnormal LVEF at follow-up (patients with LVEF <55% at follow-up (*n* = 19) had mean LVEF of 34 ± 13% at initial presentation vs. patients with LVEF >55% at follow-up (*n* = 23) displayed an initial LVEF of 47 ± 13%, *p* = 0.003).

There was no significant correlation between persistent left ventricular dysfunction and clinical presentation, inflammation on EMB, blood biomarkers or CMR parameters (LGE, T2 weighted Imaging, T2 Mapping).

### T2 Mapping in the course of disease

**Fig. 3:**
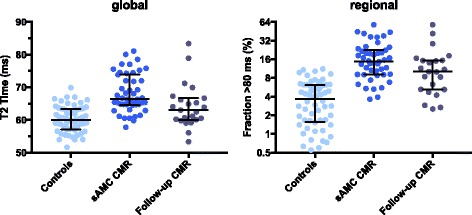
T2 time during the course of disease. Global T2 time and percentage of myocardial extent with T2 values exceeding 80 ms (Fraction >80 ms) is elevated in patients with acute myocarditis (sAMC) compared to healed myocarditis and controls. Global myocardial T2 time led to a significant distinction of controls (*n* = 60) and patients with acute myocarditis (*n* = 46) (*p* < 0.001). T2 time at follow-up examination (*n* = 23) was markedly lower (64.4 ± 6.4 ms) than in patients at acute stage of disease (*n* = 46) (68.1 ± 5.8 ms) (*p* = 0.02) and higher than in controls (*n* = 60) (60.0 ± 4.2 ms) (*p* < 0.001). Left ventricular extent with T2 time exceeding 80 ms allows a differentiation between controls (4.1 ± 3.0%) and patients with acute myocarditis (17.7 ± 11.5%) (*p* < 0.001), as well as between healed myocarditis (13.6 ± 13.3%) and controls (*p* = 0.04), but not between active and healed myocarditis (*p* = 0.14). The error bars indicate median with interquartile range. Abbreviations: CMR = Cardiovascular Magnetic Resonance, Fraction >80 ms = Percentage of myocardial extent with T2 time >80 ms

In a subset of patients (23/45) monitoring of T2 values over time was possible: Global T2 values were significantly increased at first presentation compared to follow-up examination and controls. In line with this, the extent of myocardium with T2 time above 80 ms was markedly larger in the acute phase of disease (Fig. 3).

**Fig. 4:**
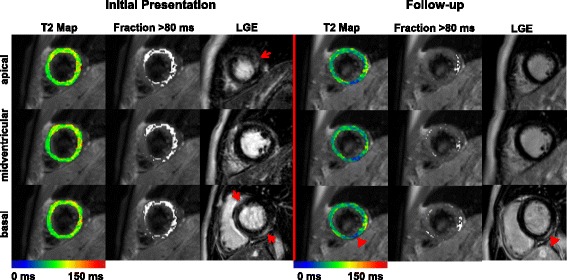
T2 Mapping and LGE at initial presentation and at follow-up. Apical, midventricular and basal short axis slices of a patient with biopsy-proven acute myocarditis are displayed at initial presentation and at 12 months follow-up. Coloured T2 maps display global T2 values in a color code ranging from 0 to 150 ms while regional T2 values exceeding 80 ms are given as white overlays. Late Gadolinium Enhancement (LGE) images are shown next to the T2 analysis in identical short axis slice. Red arrows point towards regions of LGE uptake at initial presentation. Arrowheads point towards a region with persistent LGE and with low (blue) T2 values. Abbreviations: Fraction >80 ms = Percentage of myocardial extent with T2 time >80 ms >16.8%, LGE = Late Gadolinium Enhancement

At follow-up T2 values decreased significantly in a global but not in a regional manner, however, being still elevated compared to controls (follow-up patients (*n* = 23) vs. controls (*n* = 60), global T2 time: 64.4 ± 6.4 ms vs. 60.0 ± 4.2 ms, *p* = 0.001; fraction with T2 time >80 ms: 13.6 ± 13.3% vs. 4.1 ± 3.0%, *p* = 0.004). Furthermore, patients who reached the combined endpoint or presented with persistent left ventricular dysfunction at follow-up displayed persistently elevated T2 values compared to those who recovered completely. In contrast, recovery of LVEF went along with decreasing T2 values (Fig. 4).

## Discussion

The present study substantiates the prognostic implication of CMR based myocardial T2 Mapping in patients with sAMC. This may improve individual risk stratification in this patient cohort. The core findings are: (a) Elevated global and regional T2 values in patients with sAMC predict MACE and rehospitalisation (b) Patients with normal T2 times compared to healthy controls are at low risk for adverse cardiac events (c) T2 values decline with clinical recovery of over time.

### Predictors of clinical outcome in acute myocarditis

Several studies reported that native T2 time is increased in patients with clinical diagnosis of acute myocarditis [8, 9, 19, 20]. Only recently, we demonstrated that T2 Mapping increases diagnostic accuracy for bpAMC [10].

The presented study demonstrates that measurement of global T2 time provides additional prognostic information and facilitates risk stratification in patients presenting with myocarditis. Kaplan-Meier curves generated among different groups of patients (T2 time <2 SD, >2 SD, <4 SD and >4 SD compared to controls) showed a significantly shorter event-free interval in patients with global T2 time exceeding 4 SD. Furthermore, the percentage of myocardial fraction with T2 time above 80 ms was higher in patients, who reached the combined endpoint. On the other hand patients with normal T2 values were at low risk for following events.

The fact that global T2 time at first presentation was significantly elevated in patients who experienced an adverse outcome (cardiac death, heart transplantation, ventricular assist device implantation) highlights the potential of T2 Mapping strategies to identify patients at risk. Assuming that an acute myocarditis is characterized by widespread cell damage, inflammation and edema, global T2 time yielded a higher odds ratio for predicting combined endpoint than the regional approach did. Furthermore, this study showed that an adverse clinical outcome was associated with the grade of elevation of global T2 time and amount of myocardial area with increased T2 values.

T1 Mapping provides high diagnostic accuracy in confirmation and exclusion of myocarditis and also showed its ability to differentiate between different stages of disease activity [12, 21]. Recently, Puntmann et al. substantiated the prognostic value of T1 Mapping in a large cohort of patients with non-ischemic dilated cardiomyopathy [22]. However, a prognostic value of T2 Mapping in acute myocarditis has not been demonstrated yet.

Lurz et al. demonstrated that T1 Mapping did not provide additional diagnostic information in patients with chronic symptoms, which could be explained by histological pathology [13]. Acute inflammatory responses, such as necrosis, edema and hyperemia, are believed to regress as expansion of the extracellular space due to diffuse fibrosis processes. As a result, T1 relaxation time decreases over time [21, 23]. It remains questionable, whether T1 Mapping in early stages is solely increased due to edema and hyperemia. If this is the case, T1 Mapping might suffer from decreased sensitivity compared to T2 Mapping, which could explain the lack of prognostic value. However, this remains speculative at the moment.

Recent data also confirm the value of ventricular function as an independent predictor of outcome in patients with acute myocarditis [4, 24]. Biventricular dysfunction at initial presentation has already been identified as a predictor for death and cardiac transplantation [25]. Moreover, baseline left ventricular function was a marker of prognosis regardless of the clinical pattern of disease onset [16]. The presented study yielded similar results (Table 3). The alteration of LVEF at the initial CMR was a strong predictor of outcome.

In addition, presence of LGE is a controversial predictor of outcome in acute myocarditis. Grün et al. investigated a large population of patients with biopsy-proven viral myocarditis who also underwent CMR. In their study LGE was the best predictor for all-cause- and cardiac mortality [4]. In line with this, we confirm that 91% of patients who reached combined endpoint had presence of LGE on first CMR. Note that only one patient without presence of LGE experienced the endpoint. However specificity was low (29%) rendering individual patient counselling impractical (Table 3). Similarly, Sanguineti et al. investigated 203 consecutive patients with an initial CMR-based diagnosis of acute myocarditis and described an initial alteration of LVEF as the only independent CMR predictor of adverse clinical outcome. In this context LGE was not predictive for outcome [24].

In a previous published study myocardial edema assessment by T2 weighted Imaging was a strong predictor for left ventricular function recovery [26]. Vermes et al. hypothesized that observed improvement of systolic function reflects recovery of reversible injured myocardium. Our results show that T2 weighted Imaging was neither sensitive nor specific in predicting clinical outcome. T2 weighted Imaging suffers from several limitations like motion artefacts and low sensitivity, which impair diagnostic accuracy [7, 19]. Summarizing our results did not reveal a predictive value for T2 weighted Imaging in this patient cohort.

### T2 Mapping in the course of disease

In a subgroup analysis (51% of sAMC patients), we accomplished follow-up CMR and demonstrated that T2 time was significantly elevated at initial presentation possibly reflecting acute stage of disease and declined during the healing process of myocarditis (Fig. 3). In detail, we discovered a significant decrease of T2 values in patients who experienced recovery, however being still elevated compared to healthy controls. Recently, Luetkens et al. published similar results in a small patients cohort. [27] In their study 24 patients with suspected acute myocarditis underwent several CMR examinations during follow-up. CMR markers of myocardial inflammation including T1 and T2 relaxation times demonstrated a rapid and continuous decrease over time. In the presented study, patients with on-going symptoms and persistent cardiac dysfunction displayed persistently elevated T2 values at follow-up, giving rise to a discriminative value for a CMR-based approach to distinguish patients with active myocarditis from healed stages of disease. These results might be explained by a resolution of myocardial edema in the majority of patients at follow-up examination, while those with persistent left ventricular dysfunction may still have suffered from on-going myocardial inflammation.

## Study limitations

Our CMR protocol did not include Early Gadolinium Enhancement ratio (EGEr) for complete evaluation of the Lake Louise approach. Although complete Lake Louise criteria experienced general acceptance as the reference for CMR-based diagnosis of myocarditis, it has been reported that omission of EGEr does not alter overall diagnostic accuracy [28].

Our study included a rather small sample size. However, we performed a very strict selection process according to current ESC guidelines. Furthermore 87% of patients underwent right ventricular EMB, confirming diagnosis of AMC in 55%.

Another limitation of our study is that LGE was performed eight to ten minutes after gadolinium contrast injection, which could be not the optimal timing. Recently, Rodríguez-Palomares et al. emphasized that timing after contrast injection could be of prognostic importance in patients with myocardial infarction [29].

## Conclusion

Our results suggest that assessment of myocardial T2 relaxation times at initial presentation facilitates CMR-based risk stratification in patients with acute myocarditis. Patients with normal T2 values are at low risk for following adverse cardiac events. T2 Mapping may emerge as a new tool to monitor inflammatory myocardial injuries during the course of disease.

## Abbreviations

AUC: Area under the curve
bpAMC: Biopsy proven acute myocarditis
CAD: Coronary artery disease
CMR: Cardiovascular magnetic resonance
EGEr: Early gadolinium enhancement ratio
EMB: Endomyocardial biopsy
ESC: European society of cardiology
Fraction >80 ms: Percentage of myocardial extent with T2 time >80 ms (%)
LGE: Late gadolinium enhancement
LVEF: Left ventricular ejection fraction
MACE: Major adverse cardiac events
NYAH: New York Heart Association
ROC: Receiver operator characteristics
sAMC: Suspected acute myocarditis
T2w: T2 weighted imaging
